# Quality by Design Micro-Engineering Optimisation of NSAID-Loaded Electrospun Fibrous Patches

**DOI:** 10.3390/pharmaceutics12010002

**Published:** 2019-12-18

**Authors:** Kazem Nazari, Prina Mehta, Muhammad Sohail Arshad, Shahabuddin Ahmed, Eleftherios G. Andriotis, Neenu Singh, Omar Qutachi, Ming-Wei Chang, Dimitrios G. Fatouros, Zeeshan Ahmad

**Affiliations:** 1 The Leicester School of Pharmacy, De Montfort University, The Gateway, Leicester LE1 9BH, UK; kazemuk2003@yahoo.co.uk (K.N.); prina.mehta@dmu.ac.uk (P.M.); sohail_arshad79@yahoo.com (M.S.A.); P14173154@my365.dmu.ac.uk (S.A.); omar.qutachi@dmu.ac.uk (O.Q.); 2 Laboratory of Pharmaceutical Technology, Department of Pharmacy, Aristotle University of Thessaloniki, GR-54124 Thessaloniki, Greece; andriotis@pharm.auth.gr; 3 The School of Allied Health Sciences, De Montfort University, The Gateway, Leicester LE1 9BH, UK; neenu.singh@dmu.ac.uk; 4 Nanotechnology and Integrated Bioengineering Centre, University of Ulster, Jordanstown Campus, Newtownabbey BT37 0QB, Northern Ireland, UK; mwchang@zju.edu.cn

**Keywords:** quality by design, electrospinning, fibres, oromucosal delivery, NSAID

## Abstract

The purpose of this study was to apply the Quality by Design (QbD) approach to the electrospinning of fibres loaded with the nonsteroidal anti-inflammatory drugs (NSAIDs) indomethacin (INDO) and diclofenac sodium (DICLO). A Quality Target Product Profile (QTPP) was made, and risk assessments (preliminary hazard analysis) were conducted to identify the impact of material attributes and process parameters on the critical quality attributes (CQAs) of the fibres. A full factorial design of experiments (DoE) of 20 runs was built, which was used to carry out experiments. The following factors were assessed: Drugs, voltage, flow rate, and the distance between the processing needle and collector. Release studies exhibited INDO fibres had greater total release of active drug compared to DICLO fibres. Voltage and distance were found to be the most significant factors of the experiment. Multivariate statistical analytical software helped to build six feasible design spaces and two flexible, universal design spaces for both drugs, at distances of 5 cm and 12.5 cm, along with a flexible control strategy. The current findings and their analysis confirm that QbD is a viable and invaluable tool to enhance product and process understanding of electrospinning for the assurance of high-quality fibres.

## 1. Introduction

Electrospinning is a one-step technique that yields fibres with diameters of micro- or nano-scale size. It is rapidly gaining attention in the pharmaceutical industry due to producing fibres with advantageous properties such as high porosity, flexibility, strong mechanics, and high ratio of surface area to volume [1]. Many scientists have dedicated their research to this remit with respect to the uses of these fibres, and due to their many appealing characteristics, many applications have been established [2,3]. These applications include biomedical engineering [4], imaging [5], specialised drug delivery systems [6], wound dressings and tissue engineering [7], and nano-encapsulation of bioactive compounds [8]. Technological developments to the electrospinning process have also been studied, giving rise to new ways to produce the three-dimensional (3D) fibres [9]. Examples of these include blend electrospinning [10], electrospinning using alternating current [11], and electrospinning using 3D printing technology [12]. Many more applications have been discovered, and the scope of the research towards electrospinning is continually growing [13].

**Figure 1:**
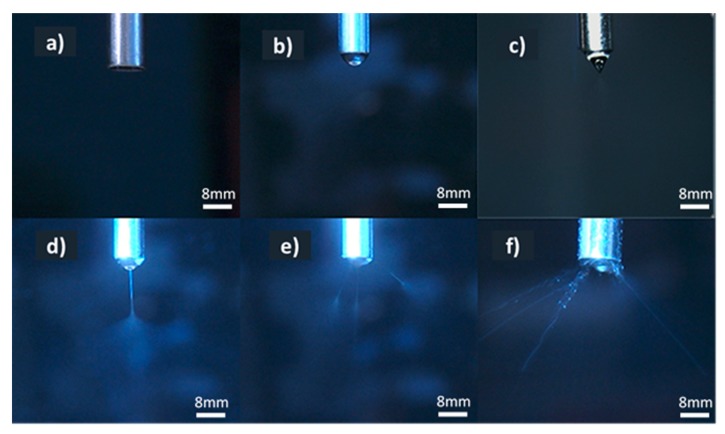
Stages of electrospinning jet formation. (**a**) Flow under no applied voltage, (**b**) droplet, (**c**) stable Taylor cone, (**d**) jet stream formation, (**e**) multiple jet streams, (**f**) dried jet streams.

The electrospinning process works by feeding a polymeric solution or melt through a capillary-like medium/needle with an applied electric field that aids in the formation of good spray patterns. Figure 1 displays the stages (a–f) of spray formation during electrospinning. Figure 1a shows what happens when the process has commenced but solution has not yet been moved to the electrospinning needle. Droplets start to emerge as the solution reaches the needle, but the applied electric field is low at this point and the solution droplets fall without undergoing electrospinning (Figure 1b). This electric field causes the solution/melt to become electrostatically charged, where the charge attempts to counter the surface tension of the solution/melt. As the electrostatic charge increases and goes into equilibrium with the surface tension of the solution, a conical-shaped droplet is formed at the tip of the needle, i.e., a Taylor cone, as shown in Figure 1c. A good understanding of the science behind the Taylor cone and the process parameters can help control its stability [14]. Once the voltage charge exceeds the surface tension value or threshold point (Figure 1d), a jet stream of the polymeric solution/melt is ejected out of the medium toward the collector, which is at ground state [15]. The self-formation of both the Taylor cone and jet stream is governed by the relationship between the coulombic repulsion interactions between ions within the solution/melt, due to the applied electric field, and the surface tension and molecular cohesion of the fluid [16]. Multiple jets can be seen in Figure 1e, which may occur when voltage is increased. This may be desirable, as multiple strands of fibre could be produced simultaneously. However, increasing the applied electric field in excess can cause the multiple jet streams to dry instantly and become stuck to the tip of the needle, as shown in Figure 1f. This would result in a reduction of fibre yield and quality. Therefore, it is essential to understand the behaviour of solutions/melts to maintain a stable jet stream that will ensure the production of good quality fibres.

Quality by Design (QbD) is a systematic approach for product and process development, where objectives are predefined and emphasis is placed on obtaining a thorough understanding of these aspects. This is achieved through sound science and quality risk management. This allows a product to be formulated and manufactured to the required safety, efficacy, and quality standards, or analysed to the required accuracy and precision on a consistent basis [17].

To start the QbD approach, the Quality Target Product Profile (QTPP) must first be defined. The QTPP may contain some critical quality attributes (CQAs). These are physical, chemical, biological, and microbiological properties of the drug product, substances, and intermediates that should be within an appropriate range to ensure the quality of the final product [18]. Risk assessments are performed to evaluate the impact material attributes and process parameters could have on CQAs and, in turn, affect the product quality. Material attributes and process variables identified as high risk are referred to as critical material attributes (CMAs) and critical process parameters (CPPs).

After completing risk assessments and all preliminary studies, a design of experiments (DoE) can be constructed with the help of statistical analytical software. It is a crucial part of the QbD approach and is used to systematically analyse and quantify relationships and interactions between the input variables and the responses of the process via multivariate analysis [19]. The design space is the “multi-dimensional combination and interaction of input variables (e.g., material attributes) and process parameters that have been demonstrated to provide assurance of quality” (ICH Q8). Working within this space is not considered a change, whereas moving out of the space is a change and would normally require a regulatory post-approval change process. The knowledge space is the space of all known aspects of the product and process. Anything outside this space will be treated as uncharted territory, where product and process understanding would be minimal. The control space is the specific area within the design space that is used for the process as normal operating requirements. This ensures the process will be kept within the design space at all times, and further assures product quality. The control space can also be a balance between the quality yield built in the product and the budget, maintenance, and production costs in industrial settings.

Continuous improvement is the final step of applying QbD, which involves constant monitoring of the CQAs, inputs, and product quality with the purpose of fine-tuning process performance to further improve quality assurance of the product, and it is continuously applied during the product lifecycle [20].

The study presented sought to implement the QbD approach to engineer electrospun fibrous structures with built-in quality for buccal administration of non-steroidal anti-inflammatory drugs (NSAIDs) indomethacin and diclofenac sodium. In this study, the analysis of indomethacin and diclofenac sodium were kept together to allow the comparison of fibres and design spaces, as both compounds are NSAIDs that are similar in size, both contain two benzene rings and are chlorinated. Furthermore, the electrospinning process is a one-step process, and there are only three excipients involved.

## 2. Materials and Methods

### 2.1. Materials

Indomethacin, diclofenac sodium and Plasdone K-90 polyvinylpyrrolidone (PVP) (high molecular weight 1.3 × 10^6^ g/mol) were purchased from Sigma-Aldrich (Gillingham, UK). Ethanol (HPLC grade) was obtained from Fisher Scientific (Loughborough, UK). Buffer pH 6.8 solution simulating saliva was prepared using NaCl (0.850 g), Na_2_HPO_4_ (0.200 g), and NaH_2_PO_4_. 2H_2_O (0.129 g) in 100 mL of distilled water. All reagents used were of analytical grade.

### 2.2. Methods

#### 2.2.1. Applying QbD

Prior to the methodology, the initial applications of QbD must first be made. With the QTTP established, risk analysis complete, and a decision made on which parameters are to be tested and controlled, a design of experiment (DoE) can be constructed. The factors to be tested and their ranges were as follows:

- Drug: Indomethacin (INDO) and diclofenac sodium (DICLO) (categorical factor)
- Voltage: 10–20 ± 0.2 kV (continuous numerical factor)
- Flow rate: 5–35 µL/min (continuous numerical factor)

The distance between the processing needle and collector was kept between 5 and 20 cm.

Since all four factors to be tested were of two levels, a full factorial design consisted of 16 runs. Additionally, four centre points were included to identify any curvature in the results, bringing the total number of runs to perform to 20. The responses that were measured were fibre diameter with an upper limit of 1000 nm, aiming to keep it minimised, and quality of fibre with a lower limit of 4, which was to be maximised. The above information was inputted into the JMP DoE dialog and the JMP software commenced simulation to create a table of experiments to perform. The simulation resulted with all possible combinations of the factor limits and centre points.

#### 2.2.2. Preparation of Electrospun Fibres

To start, 0.25 g of INDO and 4.75 g of Plasdone K-90 (PVP) were weighed and mixed together using the “doubling up” technique to make a mixture of INDO with PVP. This powder blend was then dissolved using magnetic stirring equipment in a dual solvent mixture of 80% *v*/*v* ethanol and 20% *v*/*v* distilled water to produce a 5% *w*/*v* stock formulation of INDO and PVP solution. This was repeated with DICLO.

**Figure 2:**
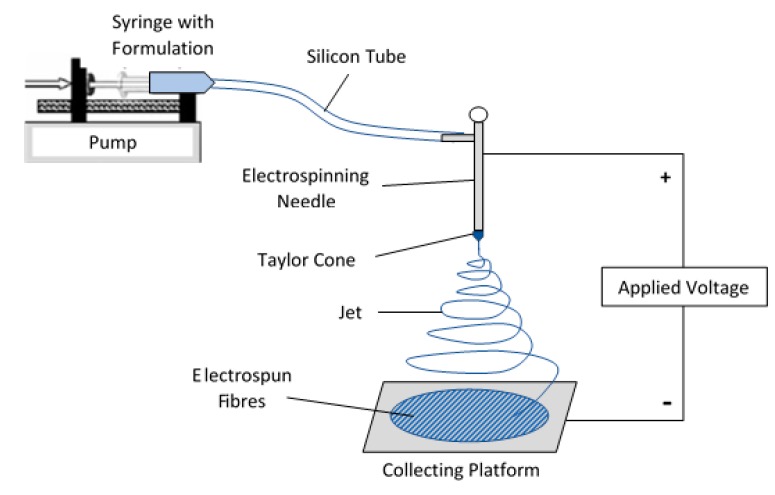
A schematic diagram showing the fundamental components of the electrospinning process.

Figure 2 shows the electrospinning equipment set-up. The flow rate of the pump was set to 20 µL/min and the distance between the conductive needle and the collection plate was 15 cm for both drug solutions. However, INDO was done at a voltage of 16 ± 2 kV and DICLO at 11 ± 2 kV. The fibres produced at this stage, alongside the stock solutions, were used for characterisation testing.

#### 2.2.3. FTIR Studies

Using a Bruker Alpha I Fourier Transform Infrared Spectrophotometer (FTIR Platinum-ATR fitted with Bruker Alpha Opus 27 FT-IR, Coventry, UK), chemical structure of raw materials and prepared fibres were analysed. An average of 30 scans at a resolution of 4 cm^−1^ was used to record the inferred spectra of each sample in the range between 4000 to 400 cm^−1^.

#### 2.2.4. DSC Studies

Perkin Elmer Jade DSC Differential Scanning Calorimeter (PerkinElmer Ltd., Shelton, CT, USA) was used to perform thermal stability tests on the prepared fibres. Each sample was weighed and placed into an aluminium pan and sealed with a punched lid. Under a nitrogen purge of 70 mL/min, samples were heated from 20 to 300 °C at a heating rate of 10 °C/min.

#### 2.2.5. TGA Studies

A thermal gravimetric analyser (Perkin Elmer Pyris 1 TGA) was used to analyse the thermal behaviour of the engineered fibres. Each sample was weighed and placed in an aluminium pan and heated at a temperature range of 20–700 °C in a nitrogen environment, using a heating rate of 10 °C/min.

#### 2.2.6. XRD Studies

X-ray diffraction was preformed to analyse the crystallinity of prepared samples using a BrukerD8-Advance diffractometer (Coventry, UK). XRD was operated at 40 kV and 40 mA, using at a scanning rate of 0.35 s/step. Sample spectra were collected in the 2θ angle from 10–50°.

#### 2.2.7. Contact Angle

Contact angle of the electrospun fibres was measured using Theta light goniometer (Biolin Scientific Attention, Stockholm, Sweden). Distilled water was micro-pipetted onto the surface of the fibrous films. Video monitor was used to assess the dynamic contact angles. The measurement was repeated three times for each sample.

#### 2.2.8. Drug loading Capacity

First, 1 mg of the samples was dissolved in 3 mL of ethanol in capped containers using magnetic stirring for 15 min. The solutions were centrifuged at 4500 rpm for 15 min, and quantification of DICLO and INDO was performed on the supernatants using UV–Vis analysis at 276 and 320 nm, respectively (UV–Vis spectrophotometer, Shimadzu, UVmini-1240, Sydney, Australia). Experiments were performed in triplicate. Drug content was calculated using Equation (1).

Drug content (% w/w) = 100 × (W_drug_/W_drug loaded fibre_) (1)

where W_drug_ is the weight of the drug and W_drug loaded fibre_ is the weight of the fibrous film used in the study.

#### 2.2.9. In Vitro Release Study

The calibration curves of INDO and DICLO in PBS pH 6.8 indicated good linearity (INDO R^2^ = 0.999, DICLO R^2^ = 0.999) in the concentration range of 1–40 ppm.

Release study of samples was conducted in capped universals in an agitating water bath at 37 °C. Then, 10 mg of every sample (fibre) was placed at the bottom of containers filled with 20 mL of PBS buffer solution pH 6.8. Samples of 1 mL were withdrawn and replaced with same volumes of preheated fresh buffer solution at predetermined time points. The samples were centrifuged at 2500 rpm for 15 min and the supernatants were analysed with UV–Vis at 320 nm and 276 nm for INDO and DICLO, respectively. Experiments were repeated four times and an average was taken. The release data profile was further analysed using the Higuchi model (Equation (2)).

Q = K_H_ t^1/2^ (2)

where Q is the quantity of drug released at time t per unit area. K_H_ is the Higuchi dissolution constant, and t is time in minutes.

#### 2.2.10. Main QbD Experiment

The same apparatus was used as in Figure 2. Each run was performed for 1 h. The first run involved processing the INDO solution at 10 kV, 5 µL/min, and at a collection distance of 5 cm. After the process was complete, the fibres were collected and stored in a labelled air-tight container for analysis. The tube, syringe, and syringe needle were replaced, and the electrospinning needle was thoroughly washed to prevent blockages from forming. This was repeated for all 20 runs and followed by subsequent scanning electron microscopy (SEM) analysis.

##### SEM Analysis

Morphology and size of the electrospun fibres from the 20 runs were studied with an electron microscope (Zeiss Evo HD-15, Cambridge, UK) where fibre diameters were measured and recorded, and the overall fibre quality was assessed using a rating system (where the fibres of poorest quality were rated 1 and those of best quality were rated 5). These results were then recorded in the DoE table, which was further simulated to obtain predictive information, such as the design space. Using the SEM images and raw results of the experiments, the distribution of fibre diameter and quality of fibre across each run were analysed for both INDO and DICLO fibres.

##### Preliminary Risk Assessments

(1) Traffic Light risk assessment for input variables

The initial risk assessment of the CQAs against the formulation variables of the process before applying controlled factors is shown in Table 1. It becomes clear that the majority of the variables pose high risks towards the primary CQAs. Therefore, some of the variables require to be controlled to avoid results being impacted.

Table 2 shows the risk scores of the CQAs against CMAs after including the well-controlled (WC) parameters, which are parameters that will be kept constant throughout the experiment. It is evident that that this greatly lowers the overall risk, as the drug used will be part of the experiment and so will be deliberately changed to see the effect this may have on the fibres to be produced.

(2) Traffic light risk assessment for process parameters

Table 3 shows the risk assessment of the CQAs against the process parameters. It can be seen that the process parameters voltage, flow rate, and distance between the needle and collector have a very high impact towards the responses (CPPs). However, these will be tested to understand the relationship between them and the primary CQAs and so will not be controlled. The same operator will be used throughout the experiment, but despite this, the possibility of human error will still be present, hence this has been left as medium risk.

## 3. Results and Discussion

### 3.1. QTTP and CQAs

To start the QbD approach, the Quality Target Product Profile (QTPP) must first be defined. The QTPP is a predefined summary of the characteristics of the product that must be of appropriate quality to meet the patient’s requirements and be fit for purpose. This helps in streamlining the process by ensuring pharmaceutical scientists and personnel of different departments (manufacturing etc.) are all working to achieve the same outcome from the beginning. Table 4 shows the summary of the QTPP of the fibres engineered here.

### 3.2. Characterisation Tests for Electrospun Fibres

All data collected here (Section 3.1) were from analysing centre point runs. The following parameters were used: Applied voltage of 15 ± 2 kV, flow rate of 20 µL/min, and working distance of 12.5 cm.

#### 3.2.1. FTIR Studies

**Figure 3:**
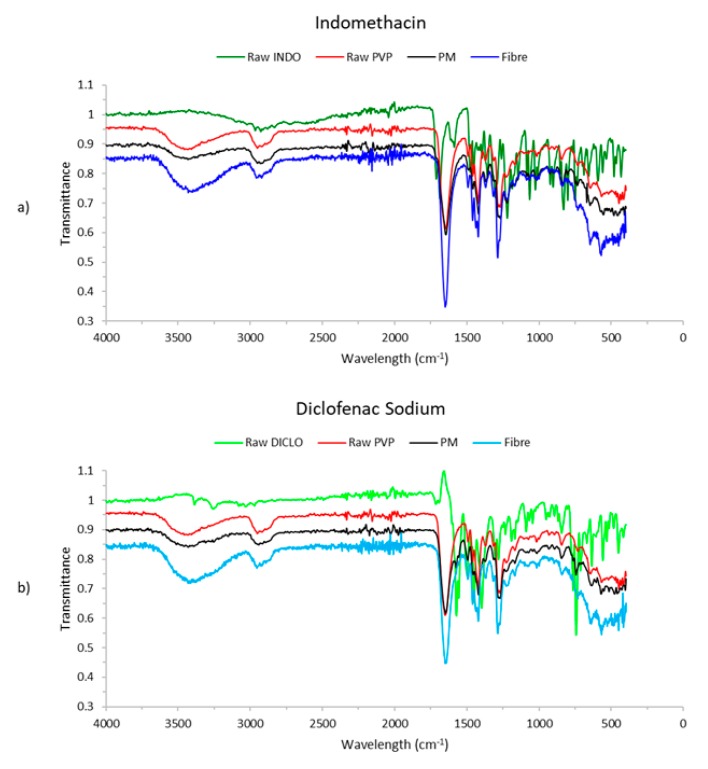
FTIR profile of (**a**) indomethacin (INDO), Plasdone K-90 polyvinylpyrrolidone (PVP), physical mixture (PM), and INDO-loaded electrospun fibres, and (**b**) diclofenac sodium (DICLO), PVP, PM, and DICLO-loaded electrospun fibres.

Figure 3 illustrates the FTIR profiles for raw INDO, raw PVP, a physical mixture (PM) of formulation (INDO + PVP), and electrospun fibre in Figure 3a, and the same in Figure 3b with DICLO.

It was clear that the two peaks at about 2900 cm^−1^ and 3400 cm^−1^ and the peaks at 1650 cm^−1^ for PM and INDO fibres were due to PVP as part of the formulation. However, no visible traces of raw INDO could be seen in PM or fibre. This may be due to a very low concentration of INDO (5% *w*/*w*) being used compared to PVP (95% *w*/*w*), where the readings for PVP could have dominated over any readings for INDO in PM and fibre. INDO fibres showed peaks of greater transmittance compared to the same peaks for raw PVP and PM, at about 1650 cm^−1^ and 3400 cm^−1^. This could be a result of INDO and PVP materials in solution interacting with each other during the process of electrospinning. The same was observed for the FTIR results involving DICLO. However, at around 1550 cm^−1^, there appears to be a very small peak that differs from raw PVP but matches with raw DICLO. This may suggest that DICLO can be identified in the PM (DICLO + PVP) and fibre via FTIR, but the observed peak was too small for it to be a reliable deduction and requires further testing for clarification.

#### 3.2.2. DSC Studies

**Figure 4:**
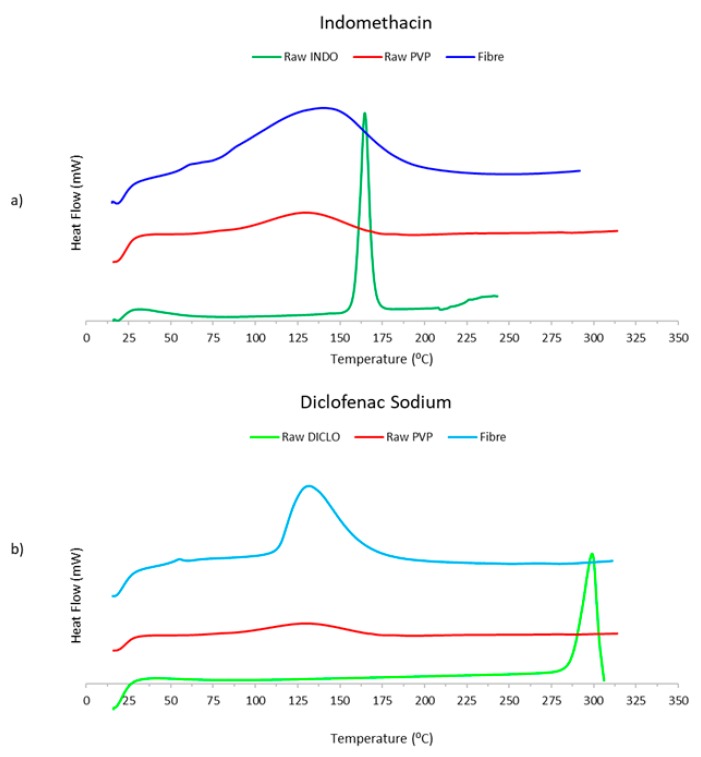
Differential Scanning Calorimetry profiles for (**a**) INDO, PVP, and INDO-loaded electrospun fibres, and (**b**) DICLO, PVP, and DICLO-loaded electrospun fibres.

Figure 4 shows the DSC results for raw INDO and DICLO, raw PVP, and INDO and DICLO fibres.

The melting points for the raw materials were indicated by the large peak, which occurs at approximately 165 °C for raw INDO and around 298 °C for raw DICLO. However, these peaks can no longer be detected within the electrospun fibre versions of INDO and DICLO. This suggests that the respective drug underwent a physical change from crystalline form to amorphous form during the electrospinning process, causing the drug to be molecularly dispersed throughout the polymeric matrix [21,22]. Furthermore, the amorphorcity of the now encapsulated drug increases surface area and hence enhances drug release and improves drug solubility, in turn increasing bioavailability. The broad endothermic peaks that start around 80 °C for raw PVP and INDO fibres and at about 112.5 °C for DICLO fibres were due to evaporation of moisture. The endothermic peaks for INDO fibres and DICLO fibres were much larger than that of raw PVP, which could have been caused by a significant increase in surface area in the fibrous structure of both drug-loaded electrospun fibres, leading to a greater extent and rate of moisture loss.

#### 3.2.3. TGA Studies

**Figure 5:**
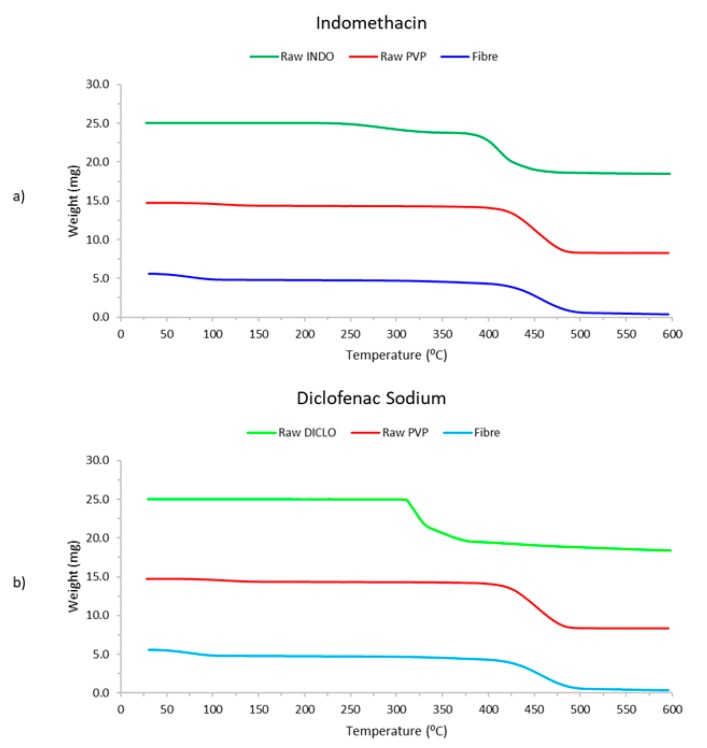
Thermogravimetric Analytical profiles for (**a**) INDO, PVP, and INDO-loaded electrospun fibres, and (**b**) DICLO, PVP, and DICLO-loaded fibres.

TGA thermograms of raw INDO and DICLO, raw PVP, and electrospun INDO and DICLO fibres are presented in Figure 5.

The profiles for raw PVP, INDO fibres, and DICLO fibres show a slight initial decrease in weight between 30 to 100 °C, which was due to dehydration of moisture from heating. The second, more distinctive reduction in weight was the result of the respective material undergoing degradation [23] from about 315–515 °C. Another observation that can be made is that the raw material peaks can no longer be detected in the TGA profile of the electrospun fibres. This corresponds to the active drug being encapsulated within the matrix of the fibre [21].

#### 3.2.4. XRD Studies

**Figure 6:**
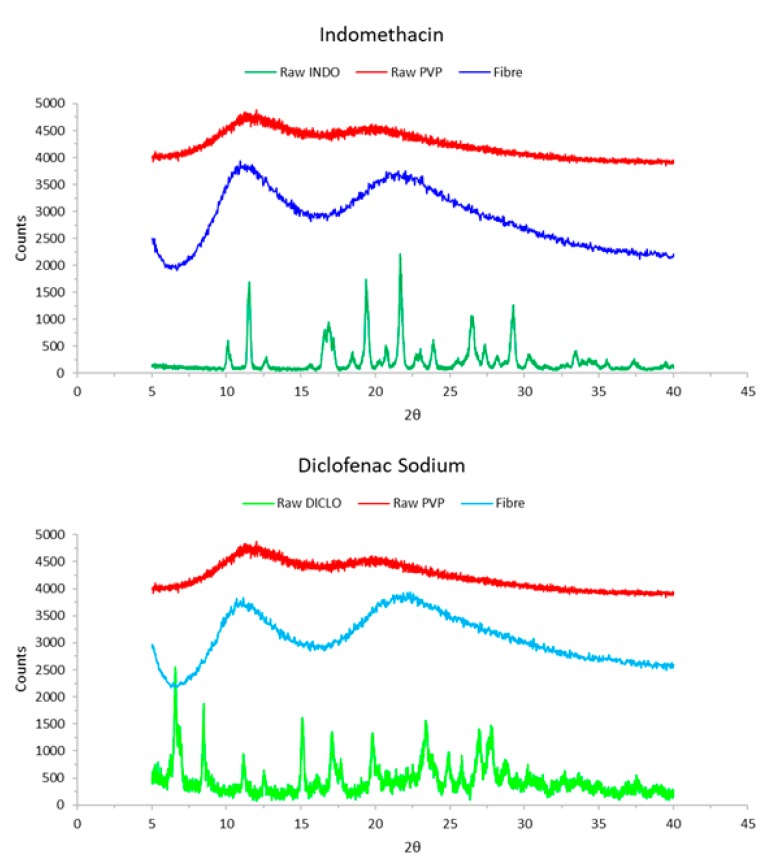
X-ray diffraction profiles of raw materials and electrospun fibres.

Figure 6 shows the XRD diffractograms of raw materials and electrospun fibres. The multiple peaks for the raw drug materials demonstrate that the drugs are crystalline in their original form.

However, these peaks are no longer visible in the fibres’ fingerprint due to the drugs converting to amorphous form and being distributed throughout the polymeric fibrous structure [24], which supports the findings realised in DSC. The peaks are also larger in the fibre profiles compared to raw PVP. This is because post-electrospinning, the crystalline peaks of the raw active materials merge with the two peaks seen for PVP, increasing the peak intensity.

#### 3.2.5. Contact Angle Measurements

**Figure 7:**
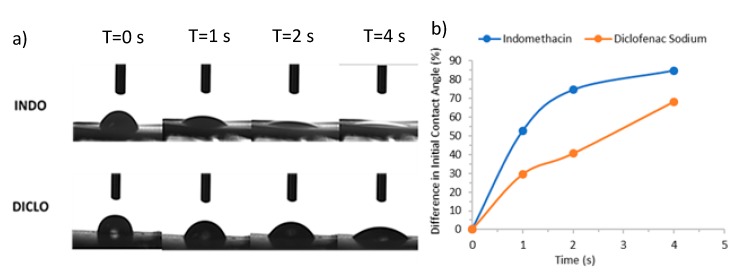
(**a**) Contact angle analysis over time for fibrous films containing either INDO or DICLO, (**b**) Mean change of initial contact angle over 4 s.

Measurements of the contact angle of distilled water on INDO and DICLO fibre films were taken at 0, 1, 2, and 4 s, as shown in Figure 7. It is clear from the results that the contact angle on INDO fibre samples decreased faster and to a lower value over 4 s from 54.55 to 8.26° compared to 88.15 to 28.01° with DICLO fibres. This suggests that the INDO fibres possessed greater wettability and hydrophilic properties than DICLO fibres [24], which may be due to differences in physical and chemical properties between INDO and DICLO.

#### 3.2.6. In Vitro Release Study and Drug Encapsulation

**Figure 8:**
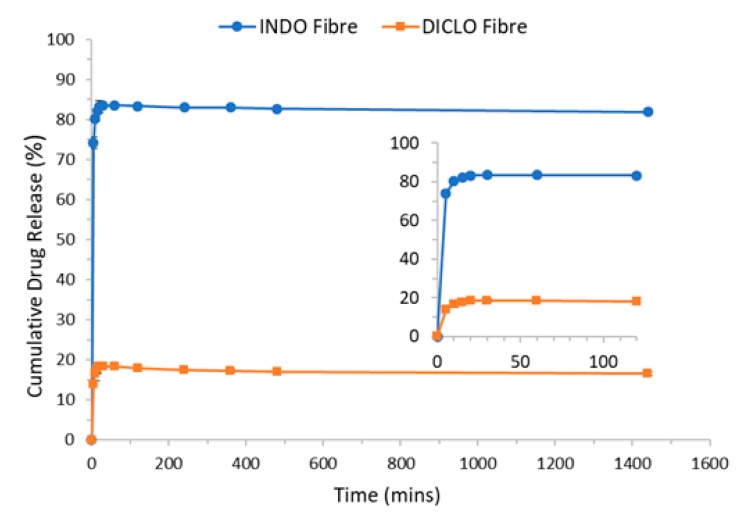
In vitro cumulative release of drug from electrospun fibres.

Fibres were found to contain 4.21% *w*/*w* of INDO post-electrospinning, while DICLO fibres contained 3.81% *w*/*w* of DICLO (Figure 8). This difference may be due to the differences in properties of the drug, such as solubility. Both fibres encapsulated less than the theoretical amount of 5% *w*/*w* of the respective drug, which is most likely due to the precipitation of the drug [24]. The electrospinning process is rapid with respect to jet forming and solvent evaporation. When the electrical field is applied, the solution is stretched at the needle exit, causing whipping and bending of the stable jet, thus forming fibres. In this process, some drug particles may precipitate onto the surface of the fibre or not adsorb onto the formed fibres, and are therefore not encapsulated in the matrix of the fibre, and hence are lost. Temporary unstable jetting may cause the formation of polymer–drug droplets which do not form part of the fibres, and are therefore lost to the surrounding environment.

#### 3.2.7. QbD Experiment

The raw results from the experiment were inputted into the DoE table, as shown in Figure S1 (Supplementary Materials). The fibre diameters were determined via SmartTiff software on SEM images, and the quality of fibre was obtained by comparing and estimating overall visual quality of the SEM image against others. It can be seen that the best results were observed in run 6 of the centre points for INDO and run 11 for DICLO. Furthermore, the centre points for both drugs gave highly desirable results, suggesting that the process parameter settings from the centre points may be the best settings to be used to obtain quality fibres for both drugs.

#### 3.2.8. Grading Quality of Fibre

**Figure 9:**
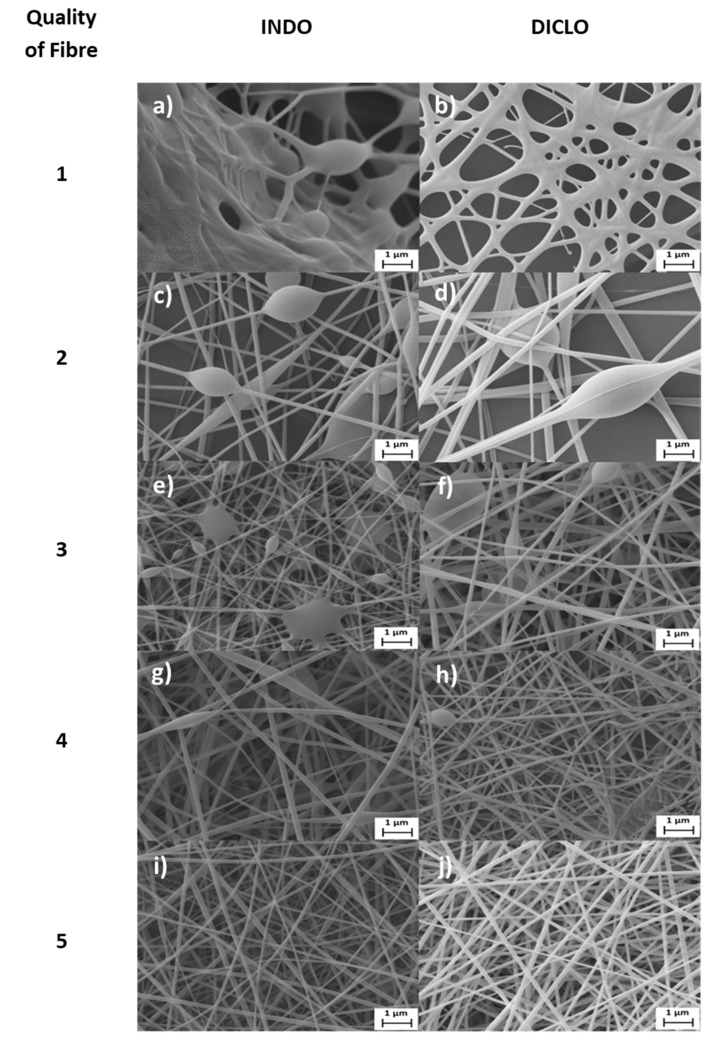
Scanning electron microscopy (SEM) images of runs using Quality by Design (QbD) experiments, showing how results were graded with respect to quality, with INDO (left column) and DICLO (right column). The runs include (**a**) run 3, (**b**) run 13, (**c**) run 4, (**d**) run 12, (**e**) run 7, (**f**) run 20, (**g**) run 9, (**h**) run 18, (**i**) run 5, and (**j**) run 11.

SEM images for post-electrospinning of INDO and DICLO solutions from the QbD experiment are shown in Figure 9, where examples of fibres with quality of fibre ratings of 1–5 are given for both drugs.

Figure 9a is the produce of INDO solution from run 3, the parameters of which can be referred to from the DoE Table in Figure S1 (Supplementary Materials), and displays very poor quality of product. This is evident from the collection of “sludge-like” mass of material that shows no fibrous quality, earning a quality of fibre score of 1. Figure 9b is a DICLO product made in run 13, which also received a quality score of 1 due to poor morphology, where the material was very damp with varying diameters. Since both runs were of the same process parameter settings, it was determined that the poor results were largely due to high flow rate and low distance. This did not allow enough time for the solution to elongate and dry into the desired fibrous form after being ejected. However, a higher voltage may have countered this, which could have increased the electrostatic interactions sufficiently enough between materials of the solution to speed up the whipping process of the solution into fibres of required quality, as proven in runs 9 and 19. In contrast, decreasing the flow rate in run 3 would not amount to much change in results, as seen in run 1, and while increasing the distance slightly improves quality of fibre, it still fails to meet the requirements. Decreasing the flow rate in run 13 would offer DICLO fibres of the highest quality, as observed in run 11, whereas increasing the distance, as in run 14, would radically enlarge fibre diameter.

Figure 9c was obtained from run 4, and Figure 9d was from run 12. Both runs offered results of low fibre density with multiple beads. Although, they consisted of some structure and the morphology did not look very damp compared to Figure 9a,b. Figure 9c shows fibre strands with lower diameters than Figure 9d, but Figure 9d seems to contain fewer beads. These balanced their differences and so both received a score of 2. Both runs had low voltage and high distance, but run 4 had a flow rate of 35 µL/min, while run 12 had 5 µL/min. However, from this alone, the reason why this occurs cannot be determined, and requires further testing and analysis to do so. From run 4, either reducing the flow rate or distance resulted in poorer fibre quality, despite lower diameters, as seen in runs 2 and 3, respectively. In contrast, increasing the voltage from run 4 produced acceptable fibres (run 10). In the case with run 12, decreasing distance reduced fibre diameter and greatly improved quality of fibre, producing the best model fibres for DICLO, as observed in run 11. Increasing the voltage helped improve fibres up to acceptable quality (run 18), but it was not as desirable, as seen in run 11. However, increasing the flow rate worsened the quality, and diameters were extremely high, as witnessed in run 14.

Figure 9e was done through run 7 and Figure 9f from run 20, both rated a quality figure of 3. However, the morphologies are very different. While Figure 9e,f shows better density of fibre compared to Figure 9a–d, Figure 9e contains multiple beads and damp, “sludge-like” areas that seem to conjoin with many strands, despite having a low overall fibre diameter. When comparing results from run 7 (Figure 9e) with run 9, it becomes clear that the flow rate plays an important role in the quality of fibre when working at the same voltage and distance of 20 kV and 5 cm. Further analysis is needed to find the reason why this occurs. A higher voltage and distance improved the quality, as revealed in run 1, which received a score of 1 at low voltage, and run 10, which received a score of 2. In the case of Figure 9f, there were no “sludge-like” areas, but fibre diameters were larger than in Figure 9e, and beads were also present. Comparing run 20 (Figure 9f) with runs 14, 18, and 19 proved that all three process parameters played significant roles in the fibre quality, where voltage and distance were the most significant. Reducing voltage would drastically reduce quality (run 14), whereas reducing flow rate (run 18) and distance (run 19) separately would improve quality.

Figure 9g was obtained from run 9 and Figure 9h from run 18. Both results show good fibre density and low fibre diameter, although Figure 9h has diameters lower than Figure 9g. However, beads are present in both and breakages in fibre are present in Figure 9h. This earns them a quality rating of 4. However, decreasing the voltage and flow rate would result in fibres of much lower quality, which was observed in runs 3 and 7, respectively. Increasing the distance from run 9 (Figure 9g) would not affect the quality, but the fibre diameter may decrease, as observed from run 10. The breakages seen in Figure 9h (Run 18) were due to the high distance, where the fibres had more time to dry, becoming brittle and causing them to break upon impact on the collector. When comparing between runs 18 and 20 (Figure 9f), the quality in run 18 was improved by reducing the flow rate. However, this could be further improved by reducing the distance as observed in run 17. Lowering the voltage was shown to greatly reduce quality of fibre (run 12).

Figure 9i,j represents model fibres exhibiting the best quality, where Figure 9i was done through run 5 and Figure 9j with run 11. The fibres have very low diameters with good consistency throughout the fibrous structure. No beads, breakage, or “sludgy” areas are present, and both show very high fibre density. Run 5 was part of the centre points of the experiment, which were performed twice, alongside run 6, where virtually the same results were achieved. With run 11, increasing the distance or flow rate greatly reduced the general quality of the fibres, as was observed in runs 12 and 13. Although increasing voltage slightly increased fibre diameter from run 11 to run 17, a model fibrous product was obtained with similarly high quality of fibre.

#### 3.2.9. Distribution of Raw Results

**Figure 10:**
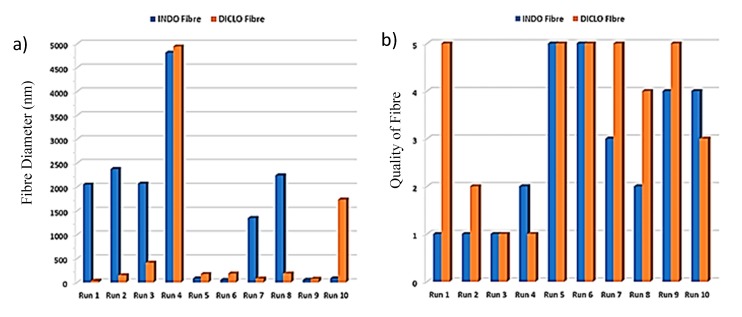
(**a**) Fibre diameter distribution and (**b**) quality of fibre across all runs.

The distribution of the raw results for fibre diameter and quality of fibre against runs is shown in Figure 10.

Each individual run from runs 1–10 for INDO consisted of the same process parameters as in runs 11–20 for DICLO, and so only runs 1–10 were kept as the range for both drugs in Figure 10 to allow comparisons to be made between them. The runs that gave acceptable fibres for INDO were found to be runs 5, 6, 9, and 10, where 5 and 6 gave the best results. Acceptable fibres for DICLO were made from runs 1 and 5–9, with the best fibres formed from run 1. In addition, it was also found that the centre point runs, runs 5 and 6, resulted acceptable fibres for both INDO and DICLO, which may suggest that the same parameters of the centre points can be used for both drugs between runs without the need to change parameter settings.

Runs 1–4, 7, and 8 produced INDO fibres that failed to meet the desired fibre diameter and quality of fibre. On the other hand, runs 4 and 10 produced fibres with diameters outside the acceptable limit for DICLO, which were also lower than the minimum required quality. However, runs 2, 3, and 10 for DICLO also formed fibres of poor quality, although the diameters were within the limits. This confirmed that the parameters of the respective runs should be avoided.

#### 3.2.10. Statistical Multivariate Analysis

**Figure 11:**
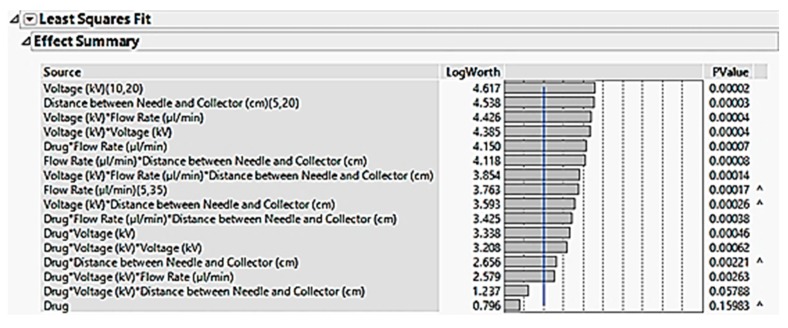
Effect summary describing the significant factors and interactions in the electrospinning process.

Prior to simulating the results, the factors were “crossed” against each other. This allowed the interactions that may have occurred during the experiment to be detected and analysed by the software. Once the simulation was completed, a list of the main factors, their possible interactions, and the level of significance they pose towards the responses was created. The significance levels were dictated by the *p*-values, where any factor or interaction with a *p*-value greater than 0.1 was considered insignificant and could therefore be removed. After removing the insignificant interactions, the leftover factors and interactions remain as significant, and so it becomes necessary to keep in order to analyse the data as accurately and reliably as possible. These factors are shown in the table in Figure 11. Although the factor “drug” has a *p*-value greater than 0.1, the *p*-values of the interactions it causes with other factors were lower than 0.1, therefore it was necessary to keep this factor as part of the simulation, as removing it would also cause the interactions involving “drug” to be removed, which would greatly affect the statistical results of the simulation.

An actual by predicted plot is shown in Figure S2 (Supplementary Materials), which describes the proximity of the actual results obtained in relation to the predicted results based on the simulation. It can be seen that there is very little spread of data and there is a graphical display of the high accuracy and reliability in the results.

The F ratio, highlighted in the lack of fit table in Figure S2 (Supplementary Materials), is the signal-to-noise ratio, which describes the robustness of the experiment. An F value close to 1 is considered to be very undesirable and the null hypothesis will be accepted, which would describe the variables as having no relationship between each other and the results obtained were purely by chance and not significant to produce model electrospun fibres. This was initially suggested to be false by the list of factors and their interactions from Figure S2 (Supplementary Materials). In addition, the F ratio calculated by the software was about 42, which is much greater than 1, further disproving the null hypothesis. This means that the four variables are related to each other and are significant to the quality and diameter of the fibres. The probability of getting an F value greater than what was calculated by chance was determined to be 2.27%, which is very low and highly unlikely to occur.

The ‘RSquare Adj value highlighted in the summary of fit of Figure S2 (Supplementary Materials) is the adjusted version of the original R square value. The original assumes that all factors and interactions in the model explain all changes in the responses, while the adjusted version has been modified to align itself to the actual results gained and describes only the factors and interactions that cause variation to occur in the responses in reality and ignores those that are very unlikely to affect the responses. Therefore, the adjusted R value would be the best estimate of the relationship between the independent variables and their interactions. However, there was not much difference between these two values, showing a good level of reliability in the model, with low experimental noise. The adjusted R square value was calculated to be about 99.8%, which is very high and shows that the process performed was very robust and the results are highly reliable. It also shows that the vast majority of the data was due to the observation and analysis of the factors and the experimental noise was negligible.

Figure S3 (Supplementary Materials) shows the actual by predicted plot, lack of fit, and summary of fit for the quality of fibre. By comparing the plots of fibre diameter and quality of fibre, it is clear that there was a much wider spread of data with the quality observed in the fibres than the diameters measured. This may be due to the process of estimating the quality being very subjective to the observer, who would then give the fibre a rating between 1–5.

No F Ratio was calculated because, unlike fibre diameter, the quality of fibre results was based on a categorical rating system rather than taking proper numerical continuous measurements.

As highlighted below, the adjusted R square value calculated was about 93.4%, which is lower than what was found for fibre diameter. In addition, there is a larger difference between the adjusted value and the original R square value. This means that a greater level of experimental noise was incorporated into the data that the software could not account for. However, since the value was above 80%, it shows that the model is still relatively significant, reliable, and robust.

### 3.3. Prediction Profilers

Prediction profilers are graphical tools that help describe the relationships and curvature between multiple variables, and also how they affect the responses as each variable is set at different parameters. Figure S4a (Supplementary Materials) shows the prediction profiler set at the maximum desirable outcome for INDO fibres. The red writing on the bottom are the parameter settings predicted to give the best fibres of high quality and those on the left are the predicted values of the responses. The grey shaded areas of the responses for the continuous factors voltage, flow rate, and distance are what the software has determined to be the error that the measurements may be susceptible to, where the predicted error ranges are displayed in brackets highlighted. The simulation predicted that the best INDO fibres will be made at 16.53 kV, 35 µL/min, and 5 cm. Since the software only works with numerical values with no units, the fibre diameter predicted at these settings was about –900, which, when scaled into the correct units, becomes approximately 0.002 nm or 2 pm (picometres). The quality of fibre was predicted to be about 5.62. This suggests that at these parameters, the fibre diameter will be extremely low and the fibre quality will be higher than what was previously observed. However, this must be verified through experiments and SEM analysis to determine if the prediction is true.

Figure S4b (Supplementary Materials) shows the prediction profiler for DICLO fibres, with parameters set to achieve the fibres of highest desirability. The parameter settings for this were approximately 15 kV, 5 µL/min, and 5 cm. The predicted responses were about –717 for fibre diameter, which scales down to about 0.004 nm or 4 pm, and a fibre quality rating of 6.75. Once again, these settings must first be tested to fully determine whether the prediction is valid.

### 3.4. The Design Space

The design space was constructed by creating a contour plot using statistical software, where six contour plots of flow rate against voltage were made. The top row shows the plots of INDO fibres, while the bottom row shows the plots of DICLO fibres. The left column shows both fibres at 5 cm distance, the middle column at 12.5 cm, and the right column at 20 cm. By displaying the contour plots in this way, the design spaces can be compared between drugs at the same distance, but also between distances of the same drug. The white space is the design space, where, when working within these parameters, the fibres produced will be of acceptable quality or higher. The red space is the boundary for fibre diameter with an upper limit of 1000 nm, but no lower limit. The blue space is the boundary for the quality of fibre with a lower limit of 4, but no upper limit. Working outside the white space and within the space of either the blue or red spaces is assumed to give poor results, leading to fibres of unacceptable quality. However, this may not be entirely true, as some areas in the coloured spaces may give acceptable results still, and vice versa, where some areas within the white design space may not offer fibres of desired quality. To improve the reliability of these contour plots, verification studies could be performed.

**Figure 12:**
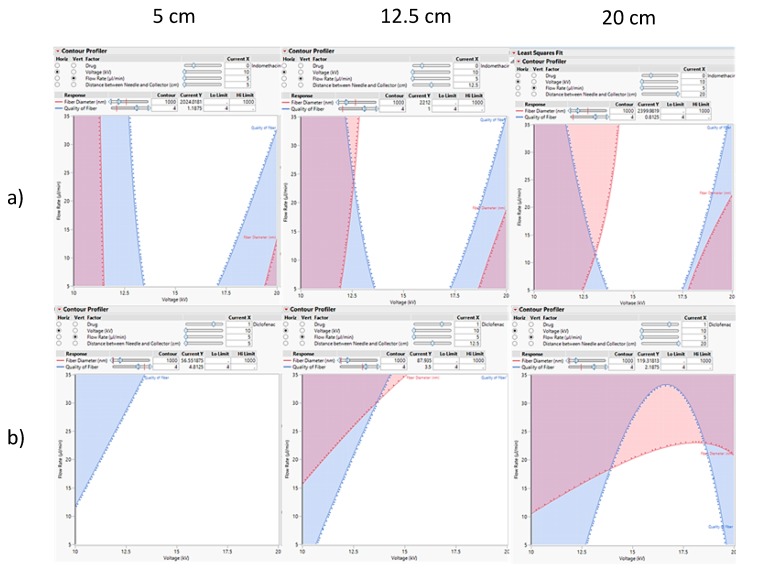
The design spaces of (**a**) INDO and (**b**) DICLO, with flow rate against voltage at various collection distances (vertical columns).

From Figure 12, it can be seen that the drug used can have a drastic effect on the design space to formulate fibres of desired quality. This may be due to differences in their physical and chemical characteristics. In addition, the distance between the needle and collector affects the design space too; however, this seems to affect the design space for DICLO fibres more so than for INDO fibres.

In general, all six design spaces are suitable to perform electrospinning of drug-loaded fibres within the respective predicted parameter ranges, separately. This is because the design space in all six scenarios is sufficiently large enough to accommodate experiments to work within the space with relative ease without risk of moving out of the space. However, if the same distance and parameters were to be used for both drugs at any time, then distances of 5 cm and 12.5 cm would be more suitable, as there would be less crossover of the design spaces by the response limits. Although, it would be more preferable to perform electrospinning at 5 cm distance for both INDO and DICLO, as the design spaces for both drugs is at their largest, giving more freedom to choose the parameter settings for the process.

## 4. Conclusions

A risk-based approach was exercised to the electrospinning of nano-scale NSAID-loaded fibres. Applying QbD, with the aid of risk assessments, characterisation tests, and multivariate statistical analysis, allowed a significantly greater understanding of the engineering process and product. This reduced the criticalities of the risks, and reliability and robustness of the process were maximised. Although all four factors assessed here demonstrated variability on the responses, voltage and distance were determined to be the most significant factors through main effects. The drug was found to be the least significant main effect; however, the interactions of the drug with other factors proved to be more significant, confirming that differing properties between drugs can vastly affect the results. All design spaces made were suitable to be used for the respective drug individually. Since all design spaces could potentially be used individually, as well as a combined design space for both INDO and DICLO fibre production, the control strategy was also highly flexible. This allows possible minor changes to be made to further assure the quality of the product. In light of the research, QbD was found to be extremely useful in promoting product and process understanding. This allows scientists and manufacturers to develop flexible design spaces and control strategies to tailor the process to their needs, and yield products of required quality consistently and reliably. It is therefore highly recommended to apply the QbD approach to all types of experiments and processes to achieve the ultimate goal of the assurance of product quality.

## Figures and Tables

**Table 1:** Traffic light risk assessment of critical quality attributes (CQAs) against formulation variables before controlling factors not being tested; red highlights high risk, yellow highlights medium risk.

CQA	Formulation Variables					
	Active Pharmaceutical Ingredient	Drug Conc.	Polymer Used	Polymer Conc.	Polymer Molecular Weight	Solvents Used
Fibre Diameter						
Fibre Quality						

**Table 2:** Traffic light risk assessment of CQAs against critical material attributes (CMAs) after applying well-controlled (WC) factors; yellow highlights medium risk, and green a low risk.

CQA	Formulation Variables					
	API (Testing)	Drug Conc. (WC)	Polymer Used (WC)	Polymer Conc. (WC)	Polymer Molecular Weight (WC)	Solvents Used (WC)
Fibre Diameter						
Fibre Quality						

**Table 3:** Traffic light risk assessment of CQAs against potential critical process parameters (CPPs); red highlights high risk, yellow highlights medium risk.

CQA	Process Parameters			
	Voltage (Testing)	Flow Rate (Testing)	Distance Between Needle (Testing)	Operator
Fibre Diameter				
Fibre Quality				

**Table 4:** The Quality Target Product Profile (QTPP) of electrospun fibrous structures.

Quality Attribute	Target
Product Form	Fibre
Fibre Diameter	≤5 µm to maximise surface area
Release Profile	Should be appropriate to supplement final dosage form
Fibre Quality	Structure and shape must be consistent throughout fibre, no breakage or droplets present
Impurities	Must be low as possible to avoid harm/instability
Pharmacokinetics	Must be appropriate
Solubility	Should be reasonable
Microbiology	Must be low and within limits
Stability	Must have consistent stability profile
Porosity	High porosity is desirable to maximise surface area and flexibility in pore size and shape
Surface Tension	Must be appropriate
Electroconductivity	Must be appropriate
Density	Must be appropriate

## Supplementary Materials

The following are available online at https://www.mdpi.com/1999-4923/12/1/2/s1: Figure S1. Design of experiments (DoE) Table highlighting the results found; Figure S2. (**a**) Actual by predicted plot for fibre diameter, (**b**) lack of fit, and (**c**) summary of fit; Figure S3. (**a**) Actual against predicted plot for quality of fibre, (**b**) lack of fit, and (**c**) summary of fit; Figure S4. Prediction profiler for (**a**) INDO Fibres and (**b**) DICLO Fibres.
