# Supplementary material for: Quality by Design Micro-Engineering Optimisation of NSAID-Loaded Electrospun Fibrous Patches
Source: Pharmaceutics. 2019 Dec 18;12(1):2. doi: 10.3390/pharmaceutics12010002 (PMC7022274; doi:10.3390/pharmaceutics12010002)
Supplement: Supplementary file 1 [file pharmaceutics-12-00002-s001.pdf]

pharmaceutics

MDPI

Supplementary Materials: Quality by Design Micro-Engineering Optimisation of NSAID-Loaded Electrospun Fibrous Patches

Kazem Nazari, Prina Mehta, Muhammad Sohail Arshad, Shahabuddin Ahmed, Eleftherios G. Andriotis, Neenu Singh, Omar Qutachi, Ming-Wei Chang, Dimitrios G. Fatouros and Zeeshan Ahmad

Project CLASSICAL FULL ...
Design 2x2x2x2 Factorial
- Model
- Evaluate Design
- DOE Dialog
- Fit Least Squares

Columns (7/0)
- Pattern
- Drug
- Voltage (kV)
- Flow Rate (µl/min)
- Distance between Needle and
- Fiber Diameter (nm)
- Quality of Fiber

	Pattern	Drug	Voltage (kV)	Flow Rate (µl/min)	Distance between Needle and Collector (cm)	Fiber Diameter (nm)	Quality of Fiber
1	1−−−	Indomethacin	10	5	5	2050	1
2	1−−+	Indomethacin	10	5	20	2374	1
3	1−+−	Indomethacin	10	35	5	2070	1
4	1−++	Indomethacin	10	35	20	4813	2
5	1000	Indomethacin	15	20	12.5	79.72	5
6	1000	Indomethacin	15	20	12.5	49.29	5
7	1+−−	Indomethacin	20	5	5	1348	2
8	1+−+	Indomethacin	20	5	20	2239	2
9	1++−	Indomethacin	20	35	5	50.65	4
10	1+++	Indomethacin	20	35	20	81.38	4
11	2−−−	Diclofenac	10	5	5	30.57	5
12	2−−+	Diclofenac	10	5	20	145.3	2
13	2−+−	Diclofenac	10	35	5	4104	1
14	2−++	Diclofenac	10	35	20	4943	1
15	2000	Diclofenac	15	20	12.5	174	5
16	2000	Diclofenac	15	20	12.5	183.4	5
17	2+−−	Diclofenac	20	5	5	77.34	5
18	2+−+	Diclofenac	20	5	20	183.4	4
19	2++−	Diclofenac	20	35	5	73.63	5
20	2+++	Diclofenac	20	35	20	1734	3

Figure S1. DoE Table highlighting the results found.

a)

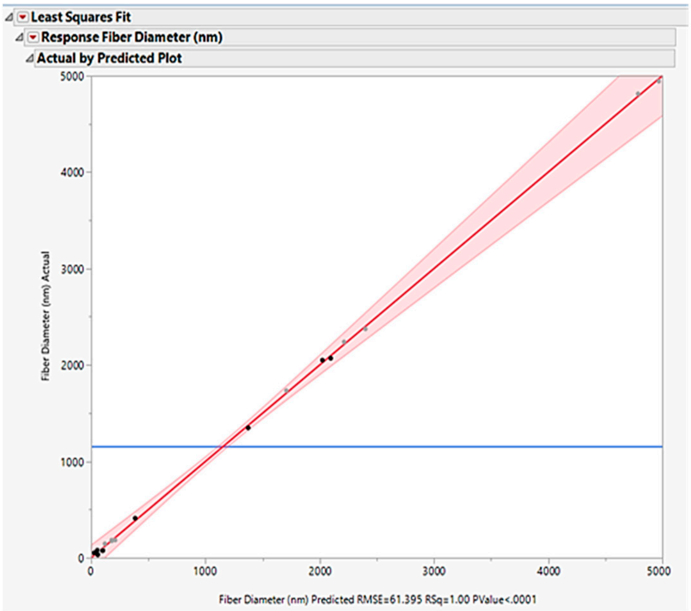

b)

Lack Of Fit

Source	DF	Sum of Squares	Mean Square	F Ratio
Lack Of Fit	1	10800.925	10800.9	42.5927
Pure Error	2	507.172	253.6	Prob > F
Total Error	3	11308.098		0.0227*
				Max RSq
				1.0000

c)

Summary of Fit

RSquare	0.99975
RSquare Adj	0.998415
Root Mean Square Error	61.39516
Mean of Response	1155.504
Observations (or Sum Wgts)	20

Figure S2. (**a**) Actual by Predicted Plot for Fibre Diameter, (**b**) Lack of Fit and (**c**) Summary of Fit.

a)

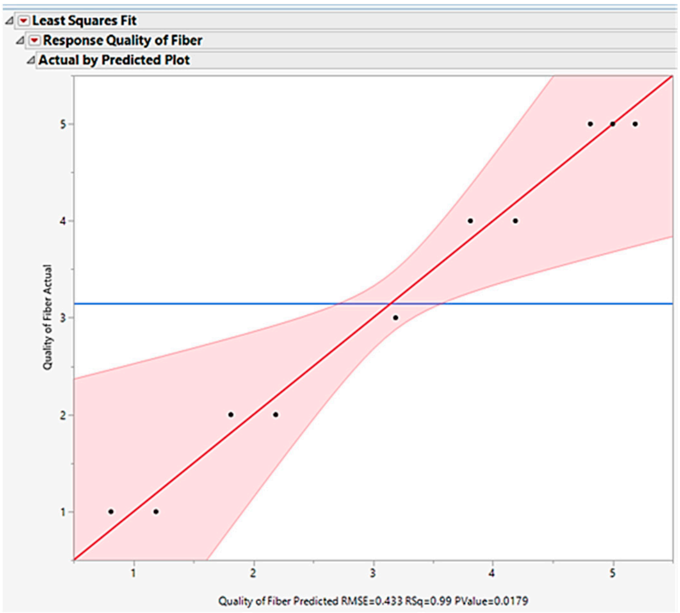

b)

Lack Of Fit

Source	DF	Sum of Squares	Mean Square	F Ratio
Lack Of Fit	1	0.56250000	0.562500	.
Pure Error	2	0.00000000	0.000000	Prob > F
Total Error	3	0.56250000		.
				Max RSq
				1.0000

c)

Summary of Fit

RSquare	0.989688
RSquare Adj	0.934693
Root Mean Square Error	0.433013
Mean of Response	3.15
Observations (or Sum Wgts)	20

Figure S3. (**a**) Actual against Predicted Plot for Quality of Fibre, (**b**) Lack of Fit and (**c**) Summary of Fit.

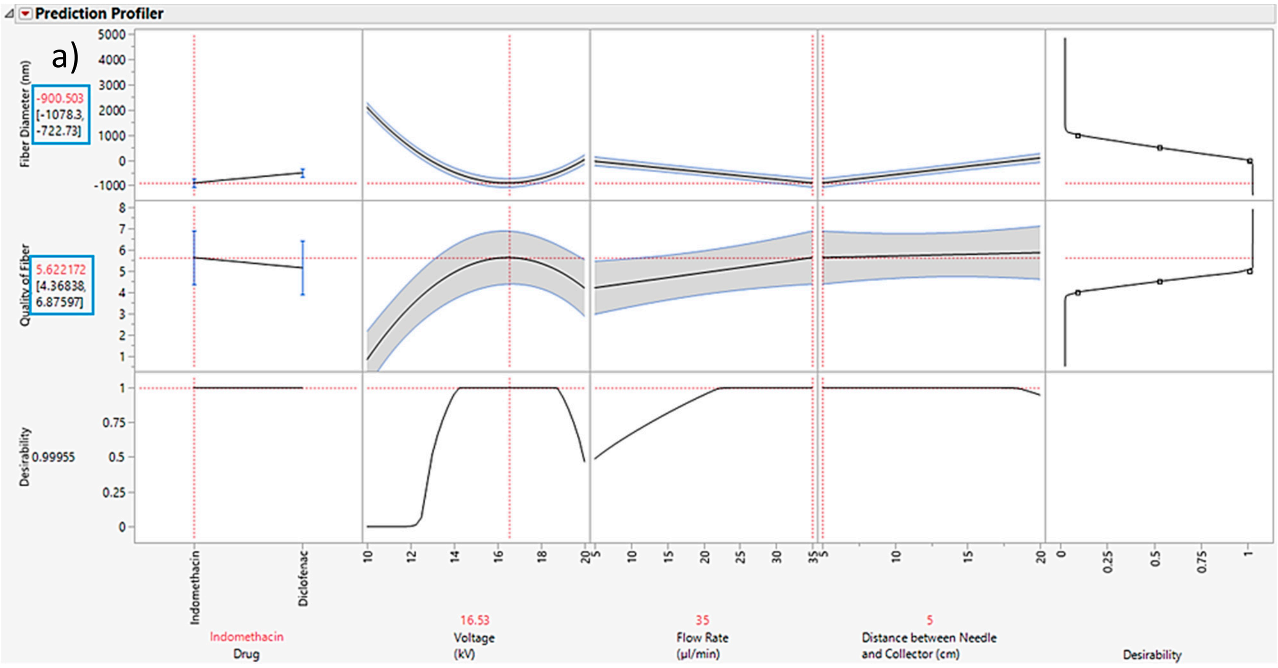

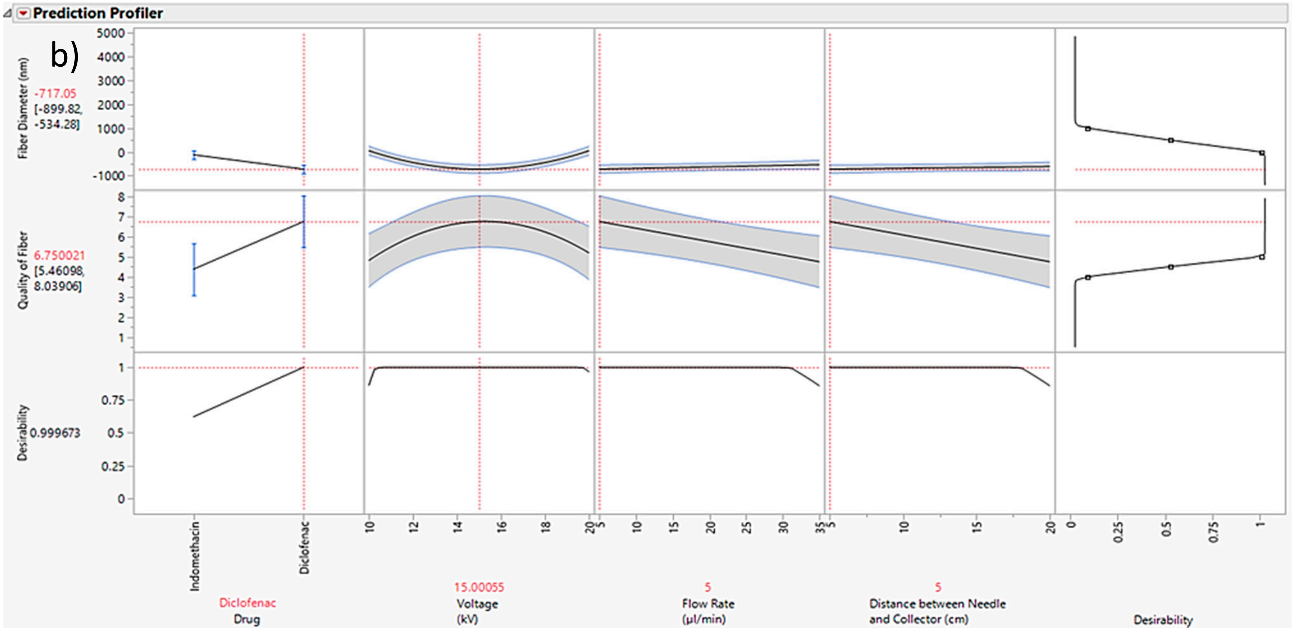

Figure S4. Prediction Profiler for (a) INDO Fibres and (b) DICLO Fibres.
